# Glycogen Synthase Kinase 3β: A New Gold Rush in Anti-Alzheimer’s Disease Multitarget Drug Discovery? Miniperspective

**DOI:** 10.1021/acs.jmedchem.0c00931

**Published:** 2020-12-21

**Authors:** Angela De Simone, Vincenzo Tumiatti, Vincenza Andrisano, Andrea Milelli

**Affiliations:** † Department of Drug Science and Technology, University of Turin, Via Giuria 9, 10125 Torino, Italy; ‡ Department for Life Quality Studies, Alma Mater Studiorum-University of Bologna, Corso d’ Augusto 237, 47921 Rimini, Italy

## Abstract

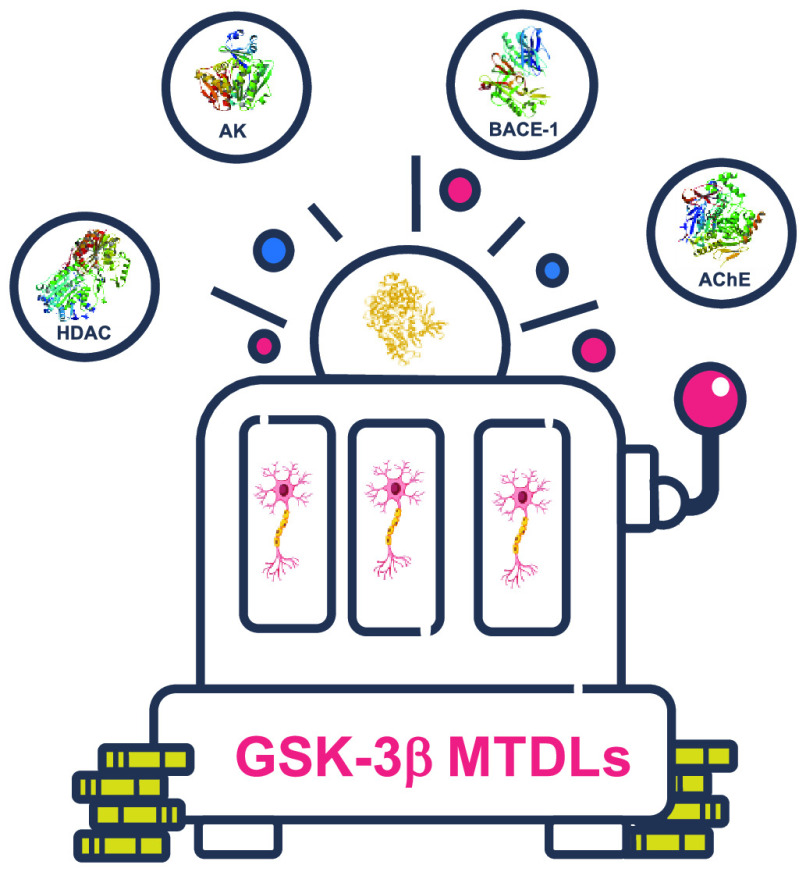

Alzheimer’s disease (AD), like other multifactorial diseases, is the result of a systemic breakdown of different physiological networks. As result, several lines of evidence suggest that it could be more efficiently tackled by molecules directed toward different dysregulated biochemical targets or pathways. In this context, the selection of targets to which the new molecules will be directed is crucial. For years, the design of such multitarget-directed ligands (MTDLs) has been based on the selection of main targets involved in the “cholinergic” and the “β-amyloid” hypothesis. Recently, there have been some reports on MTDLs targeting the glycogen synthase kinase 3β (GSK-3β) enzyme, due to its appealing properties. Indeed, this enzyme is involved in tau hyperphosphorylation, controls a multitude of CNS-specific signaling pathways, and establishes strict connections with several factors implicated in AD pathogenesis. In the present Miniperspective, we will discuss the reasons behind the development of GSK-3β-directed MTDLs and highlight some of the recent efforts to obtain these new classes of MTDLs as potential disease-modifying agents.

## 1 Introduction

### 1.1 Multitarget Drug Discovery and Alzheimer’s Disease

The drug discovery process for complex diseases, such as neurodegenerative, proliferative, or cardiovascular ones, has found renewed hope in the multitarget drug discovery (MTDD) strategy in the past decade.^1^ In fact, for diseases characterized by a multifactorial nature or therapeutic resistance developments, the classic therapeutic strategy “one molecule–one target” seems no longer appropriate and exhaustive. Such diseases arise from distress in multiple but interconnected networks that cannot be contrasted by a single drug acting on a single target.^2^

The reasons why multitarget strategy represents an attractive, concrete, and when possible, a resolutive opportunity, rely on the possibility of taking advantage of multiple additives or synergistic pharmacodynamic activities within a single molecule.^3^ To this aim, a careful target combination should be pursued. Certainly, the possibility of interacting with different targets simultaneously leads to a polypharmacological drug characterized by a more effective activity profile and fewer side effects when compared to the combined use of single-targeted drugs. Of course, this also implies greater therapeutic effectiveness and delays in the development of resistance. Moreover, polypharmacology, unlike polypharmacy, is not associated with disadvantages such as lower patient compliance and the possibility of harmful drug–drug interactions.^4^ Actually, from a pharmacokinetic point of view, the simultaneous administration of several drugs, with different pharmacokinetics, makes therapies complicated and not always applicable.^5^ Furthermore, the clinical development of a multitarget drug (MTD) offers the opportunity to save money and time since the required clinical trials are less complicated than those requested when dealing with multiple specific drugs.

However, the design of MTDs presents medicinal chemists with some challenges related to the optimization of the activity profile and physicochemical properties. In particular, MTDs are designed to obtain the same degree of *in vitro* activity for each target. Indeed, a balanced *in vitro* activity is believed to reflect the same level of target modulation also *in vivo*.^6^ Achieving similar potency (i.e., inhibiting two targets in the same concentration range) has proven to be a challenging task in many cases. Moreover, as MTDs are in most cases designed by integrating structural elements from two or more selective ligands, they are larger and more lipophilic than most commercial drugs and, as a consequence, may suffer from poor oral absorption.^7,8^

Recently, medicinal chemists moved far away from associating MTDs only by chance. In the past years, we have witnessed the growing number of papers on the drug development process of multitarget-directed ligands (MTDLs) using different strategies and technologies to obtain multiple active compounds.^9^ The different MTDL strategies provide chemical entities with the ability to hit different targets or that can bind to the same target at different binding sites related to the same complex disease, in order to modulate their activities. In light of this, the possible combinations of targets to be hit are the most varied, which makes their selection a crucial step. Undoubtedly, the accurate knowledge of the pathology to which the ligands should be directed is the base for a rational target selection.

Certainly, the MTDLs’ strategy suits perfectly to a complex pathology such as Alzheimer’s disease (AD) for which a resolutive treatment is still currently not available. AD represents the most common cause of dementia, with almost 50 million people worldwide living with this pathological condition. Together with its devastating effects on individuals, caregivers, and public health, AD represents also a major economic burden for national health systems.^10^ Decades of both public and private research have shed some light on the pathogenesis of AD, but the disease is still an enigma. Indeed, a true understanding of its onset and development is far from being achieved and many theories have been elaborated over the years.^11^ The most advanced theory concerns the amyloid hypothesis which is supported by the observation of amyloid plaques deposition in the brain.^12^ Other accepted theories include tau protein,^13^ cholinergic hypothesis,^14^ calcium homeostasis,^15^ oxidative stress,^16^ metal dyshomeostasis,^17^ inflammation,^18^ endoplasmic reticulum stress,^19^ mitochondrial disfunction,^20^ and so on. These theories have produced many potential novel drug targets.^21^ However, despite massive investments and the countless number of molecules going to clinical trials every year amid enthusiastic expectations, unfortunately and disappointingly, no new drug has entered clinical practice in Europe and U.S. since memantine’s approval.^21^ Since memantine obtained FDA approval in 2002, AD could be considered, without any doubt, the “black hole” of drug discovery, being the pathology with the highest attrition rate. Recently, in late 2019, China’s National Medical Product Administration (NMPA) has conditionally approved sodium oligomannate, a mixture of acidic linear oligosaccharides derived from marine brown algae, for the treatment of mild to moderate AD.^21,22^ Its full mechanism is not completely known, but evidence shows that it remodels gut microbiota and induces anti-inflammatory effects.^22^

On the basis of the cholinergic hypothesis and the discovery of acetylcholinesterase (AChE) inhibitors, different therapeutic strategies have emerged to delay or reverse the devastation of AD.^23^ Despite efforts, the only commercially available therapies for AD are represented by AChE inhibitors and the NMDAR antagonist memantine. However, none of these drugs are able to mitigate neuronal loss or reverse cognitive impairments or to act as a truly disease-modifying drug.^24^

It is a current belief that a single compound capable of fulfilling a scenario as intricate as that which characterizes a multifactorial disease should have a more significant impact on the course of disease progression. Since the advent of the MTDLs strategy, an increasing number of papers have been published on the discovery of anti-AD drug, and as expected, AChE has maintained its popularity as an AD-related target also in the MTDLs era. Indeed, a large part of the MTDLs strategies adopted in AD are based on the combined inhibition of AChE and another AD-relevant target.^25^ However, since Cavalli, Bolognesi, and co-workers brought to light the first-in-class dual β-secretase (BACE-1)/glycogen synthase kinase 3β (GSK-3β) inhibitors, the interest in developing MTDLs acting as GSK-3β inhibitors has increased.^26,27^ Undoubtedly, this is also a consequence of the growing importance of the tau hypothesis and the prominent role assumed by GSK-3β in this context.^28^

#### 1.1.1 Tau Hypothesis

Tau is a soluble microtubule-binding protein whose main role is to stabilize microtubules in axons to direct axonal transport and cytoskeletal growth. In AD and in other tauopathies, such as frontotemporal dementia, progressive supranuclear palsy, and so on, tau becomes hyperphosphorylated and deposits in insoluble aggregates. In normal condition, tau is highly hydrophilic, while abnormal hyperphosphorylation is the most compelling cause of tau dysfunction.^29^ Hyperphosphorylated tau and aggregates are not able to bind to tubulin and promote microtubule assembly causing their disruption.^30,31^ However, other alterations such as conformational changes^32^ and truncation of tau^33^ have also been implicated in AD pathogenesis. Several molecular mechanisms may be underlying the toxicity associated with tau including disruption of calcium homeostasis^34^ and caspase activation.^35^ Tau accumulation causes extensive damage to the transport and signaling systems, cytoskeleton, and mitochondria.

Different forms of tau have been observed in AD, such as dimer/trimer and small soluble oligomers, which are not always phosphorylated, as well as filaments and neurofibrillary tangles (NFTs) that are always phosphorylated. Although NFTs are considered one of the two hallmarks of the disease, recent evidence suggests that other forms of tau may be more toxic than NFTs, such as small soluble tau oligomers.^35−38^ This was also observed for other AD-related proteins like Aβ and α-synuclein,^39^ where soluble species were in fact the most toxic.^40,41^ In addition, some reports even discuss a possible protective role for NFTs.^35^ Of course, more studies are still necessary to confirm these findings.

Even if tau and tangles are not specific for AD, the correlation between cognitive dysfunctions and the localization of tangles present in this neurodegenerative disorder is very strong.^42^ Intraneuronal tangles containing hyperphosphorylated tau are a well-known hallmark of AD pathology, along with senile plaques containing extracellular amyloid-β (Aβ).^43^ Aβ is physiologically produced even if its role in normal brain is not completely understood. However, in AD, a serious imbalance between its production and clearance leads to the formation and accumulation of oligomers, filaments, fibrils, and ultimately, plaques. Identification of the true toxic species has been tricky, but the experimental evidence now points to oligomeric and β-sheet-rich fibrillar aggregates as responsible for the toxic effects mediated by Aβ.^44^ Aβ induces toxic effects through different mechanisms.^45^ For instance, Aβ accumulation in the brain leads, among others, to loss of synapses and changes in neuronal activity and synaptic transmission.^46^ Aβ peptide has been involved also in metal-mediated oxidative stress,^47^ and Aβ aggregates were able to inhibit telomerase activity both *in vitro* and *in vivo*.^48^

Despite the uncertainty about the relative roles of Aβ and tau in AD, the stronger correlation between NFTs and memory impairment suggests the existence of a closer connection between tau pathology and neurodegenerative events than those observed with Aβ aggregates.^49^ Moreover, since tau mutations responsible for some frontotemporal dementia were identified,^50^ it was also possible to generate transgenic models showing severe tau pathology.^51^ This aspect is of crucial importance for the demonstration of *in vivo* pharmacodynamic effects of tau-based drugs.

Different strategies can be explored in the search for anti-tau therapies, even though most approaches currently in clinical trials are immunotherapy-based.^52^ Regarding small molecules, the two therapeutic strategies that can be followed can be summarized in (a) inhibition of hyperphosphorylated tau aggregation and (b) blockage of tau hyperphosphorylation. Although inhibition of tau aggregation appears more attractive, due to the role of tau aggregates in the development of the pathology, many challenges presented by antiaggregation approaches^53−55^ have led to the reduction of tau hyperphosphorylation as the most suitable strategy to be pursued.^52^ Indeed, the discovery of protein–protein interaction (PPI) modulators proved to be very difficult mainly due to the lack of a defined binding site. In particular, the interactions between proteins frequently take place over relatively large and flat surfaces. Moreover, in most cases there are unknown natural small ligands that can be used as starting point for a drug discovery campaign. Similarly, high-throughput screening (HTS) is not particularly suitable since combinatorial libraries often lack chemical scaffold adapted for discovery of PPIs modulators.^55^

The extent of tau phosphorylation is increased in the brain of patient with AD.^56^ The number of characterized hyperphosphorylated sites is relatively small. Around 40 serine/threonine phosphorylation sites have been characterized.^49^ The phosphorylation at these sites can promote different functions regarding the enhancement of tau fibrillization,^57^ the reduction of tau binding to microtubules,^58^ and the prevention of tau aggregation.^59^

Therefore, concerning the blockage of tau hyperphosphorylation, the main obstacles encountered in adopting this strategy are related to the identification of the kinase to be targeted and the selection of suitable inhibitors. Regarding possible targeted kinases, cyclin dependent kinase 5 (CDK5) and GSK-3 are the most relevant according to *in vitro* studies evaluating tau phosphorylation.^60,49^ Kinase inhibitors are employed in many clinical fields, particularly in cancer treatments.^61^ However, selectivity is the main problem related to the development of a kinase inhibitor, since the vast majority of these molecules bind to their target ATP-binding site.^62^ All of the approximately 518 kinases of the human kinome use ATP as substrate to transfer the phosphate group to different amino acids, mainly Ser, Thr, and Tyr. Therefore, the ATP-binding site contains many conserved regions and features that are essential for substrate recognition and catalysis. Most of the kinase inhibitors available are competitive with ATP for its binding site and, therefore, establish key interactions with amino acids highly conserved within all of the kinome. As consequence, such molecules suffer from low selectivity, which could be responsible for off-target toxicity. Selectivity can be enhanced in two different ways by exploiting (a) the few distinct pockets and residues located in the vicinity of the ATP binding site that are not used by ATP which characterize each kinase or (b) the highly specific substrate or allosteric sites.^63,64^ It is clear that selective inhibitors, although they have often been characterized by lower affinity compared to ATP-competitive inhibitors, have the significant advantage of lower off-target toxicity.

Another challenge in the drug discovery process associated with CNS diseases such as AD is the overcoming of the blood–brain barrier (BBB). Molecular weight (MW), lipophilicity (log *P*), polar surface area (PSA), as well as interaction with P-glycoprotein efflux transporter (P-gp) influence the BBB penetration capacity of a molecule. Chico et al. reported that kinase inhibitor drugs tend to have higher mean values for these parameters when compared to other known CNS-penetrating compounds.^65^ For instance, imatinib fails a glioma trial because of its poor brain uptake due to its high MW and PSA values, which are greater than those of other BBB-penetrant drugs.^66^ Furthermore, imatinib is also a P-gp substrate.^67^ On the other hand, dasatinib is characterized by higher MW and PSA compared to imatinib but does not seem to be a substrate of P-gp.^68^ However, it is very difficult to come up with a general rule due to the complex nature of the *in vivo* absorption and when different physicochemical characteristics have to be taken into consideration.

### 1.2 Glycogen Synthase Kinase 3β (GSK-3β): Structure and Functions

GSK-3 is a highly conserved serine/threonine kinase ubiquitously expressed and constitutively active in unstimulated tissues. Although it was initially characterized as a cytosolic protein kinase, some nuclear functions were also reported.^69^ The genes encoding this kinase have been identified in every investigated eukaryotic genome. In particular, *GSK-3A* and *GSK-3B* are the two genes that encode GSK-3 in mammals. Specifically, these genes encode two proteins of 51 and 47 kDa whose domains show a very high homology (98%) but differ within their N- and C-terminal regions. Indeed, the 4 kDa difference in the protein masses is due to the presence of a glycine rich region at the N-terminal domain of the α isoform. The amino-terminal lobe is predominantly composed of β-sheets, while the C-terminal lobe is mostly α-helical. The ATP binding sites can be considered essentially identical, making remote the possibility of identifying isoform-selective inhibitors.^70^ However, recently significant advances in the field of isoform-selective inhibitors have been achieved by exploring small difference in the hinge region (Asp133 → Glu196 switch) to discover paralog-selective inhibitors.^71^ GSK-3 was first identified as the kinase that phosphorylates and inhibits glycogen synthase (GS), the rate limiting enzyme in glycogen synthesis.^72^ Prior to GSK-3 phosphorylation, GS is prephosphorylated on a residue located at four amino acids C-terminal to the GSK-3 phosphorylation site, representing a frequent consensus sequence (S/TXXXS/T) for GSK-3 phosphorylation.^73,74^ Many other substrates are phosphorylated by GSK-3 responsible for regulating other functions such as cell growth and survival, cytoskeletal organization, immune responses, circadian rhythm, and development.^72^ Numerous substrate proteins are functionally inhibited after being phosphorylated by this kinase.^75^

The mechanisms responsible for controlling GSK-3 activity are very complex and depend on specific signaling pathways. Several protein kinases, such as the protein kinase B (PKB/Akt), cyclic AMP-dependent protein kinase (PKA), and atypical protein kinase C (PKC), are capable of phosphorylating and inactivating GSK-3 at the N-terminal domain site. Among these proteins, PKB/Akt is able to inactivate the kinase through phosphorylation in response to insulin.^76^ What turns GSK-3β into an appealing target for neurodegenerative disease is its deep implication in the Wnt-β-catenin signaling pathway, profoundly implicated in embryonic development and human homeostasis.^77^

In this signaling pathway, Wnt blocks β-catenin phosphorylation leading to transcription of target genes. On the opposite side, phosphorylated β-catenin is recognized by ubiquitin and targeted for proteasomal degradation. The main role of GSK-3 is to keep β-catenin levels low by phosphorylating it. However, the mechanisms of GSK-3 regulation in this pathway are not completely clear.^78^ This signaling pathway plays a crucial role in neuronal development and in adult central nervous system (CNS) physiology.^79^ In particular, it regulates neurite outgrowth in adult, synapse formation and plasticity and neurogenesis.^80^ GSK-3 antagonizes this signaling pathway, while a pharmacological inhibition of the enzyme activates this pathway and stimulates neurogenesis.^79^

The involvement of GSK-3 in different pathways clearly explains why this enzyme can be considered an important cellular nexus able to integrate several signaling systems, second messengers, and cellular stimulants.

### 1.3 The Role(s) of GSK-3β in Alzheimer’s Disease

**Figure 1:**
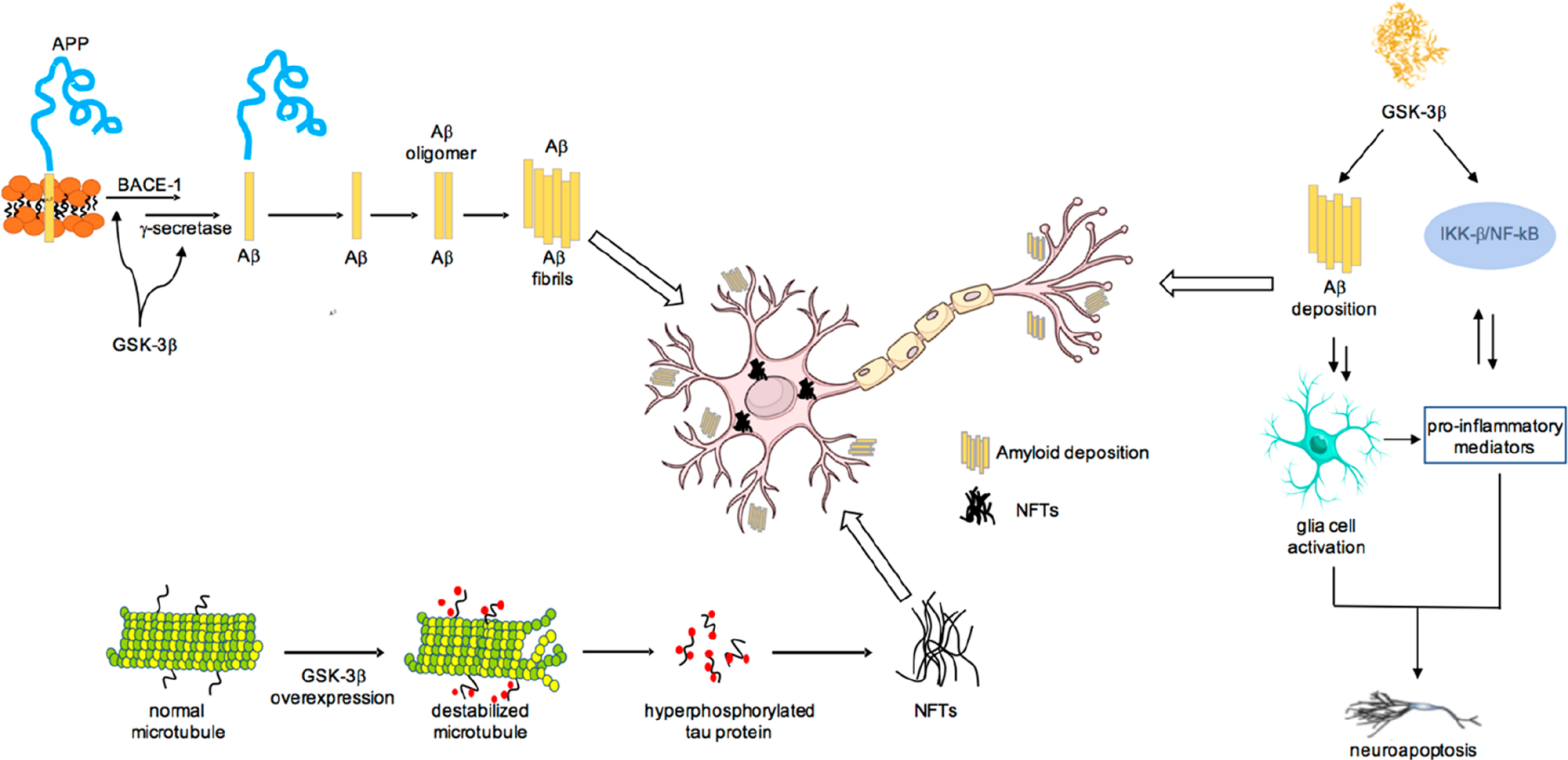
Involvement of GSK-3β in AD results from many activities. The most significant are reported in this figure. GSK-3β contributes to amyloid deposition production affecting the function of presenilin 1 (PS1) and the enzymatic cleavage of APP mediated by BACE-1; senile plaques are derived from the abnormal extracellular accumulation and deposition of Aβ peptide. GSK-3β is responsible for the hyperphosphorylation of tau protein and, as consequence, NFTs formation. GSK-3β is involved in neuroinflammation promoting the production of cytokines in astrocytes and microglia.

In the CNS, GSK-3β is the most abundant isoform and its expression levels are known to increase with age.^81^ The activity of GSK-3 is crucial for cellular signaling and to control brain functions related to development, metabolic homeostasis, neuronal growth, and differentiations, as well as cell polarity, fate, and modulation of apoptotic potential.^82−85^ GSK-3β is found to be hyperactivated in the brain of AD patients, and compelling evidence supports that it is the main tau kinase involved in AD’s pathology (Figure 1).^28,86^ As mentioned above, it is responsible for the hyperphosphorylation of tau protein, an important component of NFTs, which confers it a key role in the pathogenesis of AD.^87^ Indeed, the tau’s affinity for microtubule depends on its phosphorylation status and GSK-3β-mediated hyperphosphorylation leads to microtubule disassembling and NFTs formations.^28^ There are plenty of amino acid residues that are phosphorylation targets, and these sites are mainly close to microtubule binding domains where PPI take place. Consistently, several reports indicated that GSK-3β inhibition reduced tauopathy and degeneration *in vivo*.^88^

GSK-3β is also involved in Aβ-induced toxicity through different mechanisms.^89^ Aβ is obtained from a series of proteolytic cleavage of amyloid precursor protein (APP), a transmembrane protein highly expressed in the brain, which undergoes two different metabolic pathways mediated by a group of secretases.^90^ The nonamyloidogenic pathway mediated by α-secretase leads to fragments easily degraded, while the amyloidogenic pathway, mediated by BACE-1 and γ-secretase complex, leads to the formation of Aβ peptide which accumulates in deposits in AD brain. In particular, APP is cleaved by BACE-1 producing soluble sAPPβ and a fragment, called C99, which is further cleaved by the γ-secretase complex generating APP intracellular domain and Aβ (Figure 1). GSK-3β regulates Aβ production affecting the function of presenilin 1 (PS1),^91^ a component of the γ-secretase complex, and the enzymatic cleavage of APP mediated by BACE-1.^92^ Furthermore, NF-κB, overexpressed in AD patients, mediates GSK-3β-induced BACE-1 expression.^93^ To complete this loop, it has been observed that Aβ blocks Wnt-mediated GSK-3β-inhibition leading to an increase in Aβ formation and tau hyperphosphorylation.^94^

Further, GSK-3β is expressed in both microglia and astrocytes where it promotes production of cytokines, such as IL-1, IL-6, and TNF-α and may contribute to the development and progression of neurological disorders, such as AD, by regulating the neuroinflammation process.^18,95,96^

Compelling evidence also indicates that GSK-3β plays a critical role in synaptic plasticity and memory formation.^84,85^ Indeed, GSK-3β phosphorylates and regulates the function of an impressive number of transcription factors that play critical roles in neuronal plasticity such as NF-κB,^97^ heat shock factor 1 (HSF1),^98^ MYC,^99^ and cAMP response element-binding protein (CREB).^100^ Furthermore, GSK-3β is a critical regulator of the balance between long-term potentiation (LTP) and tong-term depression (LDP).^85^ In particular, it has been observed that LTP induction prevented LDP via GSK-3β inhibition and that GSK-3β inhibitors blocked the induction of LTD.^101^

GSK-3β also downregulates β-catenin signaling that influences synaptic size and strength. Indeed, it phosphorylates β-catenin leading to its proteasome degradation.^102^ Furthermore, overexpression of GSK-3β impairs the hippocampal neurogenesis in adult. GSK-3β is also involved in the degeneration of neurons due to the hyperactivation of NMDA receptors and consequent intracellular calcium accumulation. The activation of NMDA current is also modulated by Aβ oligomers leading to cell death.^103^

### 1.4 GSK-3β Inhibitors

Kinases represent key nodes at the intersection of multiple intracellular pathways, and deregulation of their activity has been implicated in various pathologies. For this reason, kinases have been intensively investigated as drug targets and 52 kinase inhibitors have been approved by the FDA.^104^ These drugs target nearly 20 different kinases, but most of them are used for the treatment of proliferative diseases.^104^ Recent evidence highlights that CNS protein kinases are emerging as important therapeutic targets in AD.^65^ GSK-3β is probably the most known kinase involved in AD. At the same time, increasing significance is being attributed to death-associated protein kinase 1 DAPK1,^105^ p38α mitogen-activated protein kinase MAPK,^106^ PKA, PKC, Rho-associated protein kinase 1 ROCK1,^107^ and FYN^108^ as targets for neurodegenerative disorders.

GSK-3β inhibitors have several chemotypes and include small cations and organic compounds, both synthetic and isolated from natural sources. Lithium was the first GSK-3β inhibitor used in clinical practice to treat bipolar disorder and major depression.^109^ Lithium prevents Aβ-induced toxicity and tau phosphorylation both *in cell* and *in vivo* models of AD and improve cognition in transgenic mice.^110^ Different clinical trials have produced positive results; in patients with mild cognitive impairment (MCI), long-term treatment with lithium significantly reduced phospho-tau levels in cerebrospinal fluid and improved cognitive parameters.^110^ Another study conducted with microdoses of lithium for 15 months resulted in successful decrease in cognitive decline in AD patients.^111^

**Figure 2:**
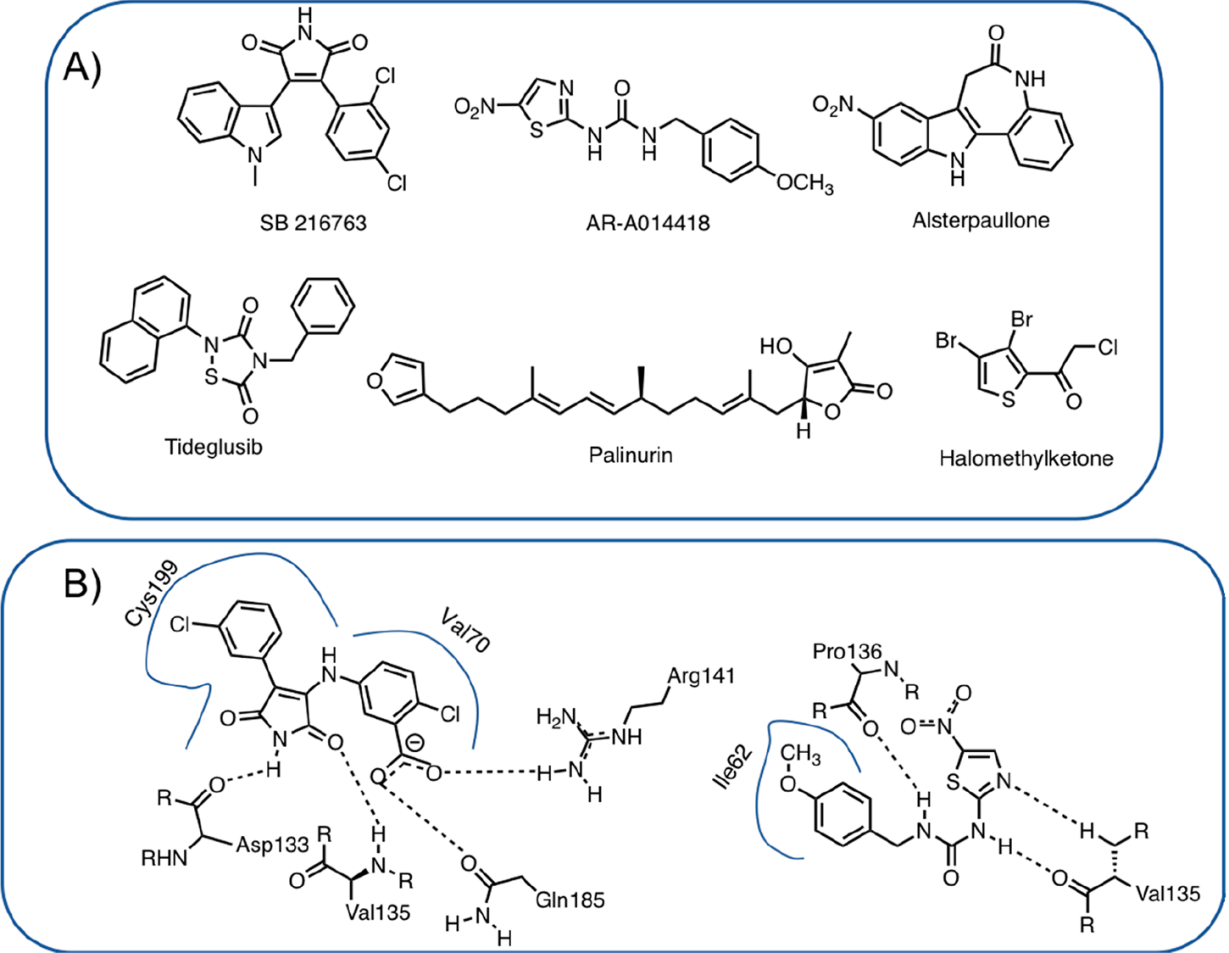
(A) Structure of selected GSK-3β inhibitors. (B) Schematic interactions of a maleimide-based GSK-3β inhibitor and AR-A014418 within the ATP-binding site (PDB codes 1Q4L and 1Q5K).

Organic GSK-3β inhibitors may be of natural or synthetic origin and, in general, are very different in structure, covering a wide range of chemical spaces. Plenty of excellent reviews have been published concerning these inhibitors, and readers are referred to them for in-depth discussions.^112,113^ On the basis of their mechanism of inhibition, they are commonly classified as ATP-competitive or non-ATP-competitive. Most inhibitors belong to the first group and act by blocking the enzyme competing with ATP for its binding site. These classes of compounds are often characterized by a very high affinity, usually in the nanomolar range of concentrations. Selectivity over other kinases represents one of the main issues associated with these compounds. Plenty of ATP-competitive inhibitors have been cocrystallized with GSK-3β and the complex structures solved by X-ray crystallography; therefore, the design of novel, highly selective ATP-competitive inhibitors may be achieved because of structure-based methodologies. Concerning ATP-competitive inhibitors, several maleimide-based inhibitors have been synthesized with a high degree of chemical diversity: linear, macrocyclic, or metal-based.^112^ The maleimide scaffold establishes key H-bonds with amino acids located within the hinge region; in particular, the nitrogen atom interacts with the carbonyl oxygen of Asp133, while one of the two carbonyl oxygens interacts with the backbone nitrogen of Val135 (Figure 2B).^114^ Other ATP-competitive inhibitors are based on the structure of thiazolylureas, such as AR-A014418.^115^ It inhibits GSK-3β with a *K*_i_ of 38 nM, it does not inhibit related kinases, and it is able to block tau phosphorylation. Crystal structures have been obtained, and AR-A014418 binds to the hinge region via three hydrogen bond interactions (Figure 2B).^115^ Paullones, such as alsterpaullone, are another interesting class of ATP-competitive inhibitors.^116^ The crystal structure revealed that alsterpaullone established H-bonds with Val135 while the nitro group established H-bond interactions with Lys85.

Selectivity can be obtained with molecules that interact with specific sites of a given kinase, such as substrate binding domain or allosteric sites. Among the different compounds, tideglusib, reported in 2002 by Martinez et al.,^117^ and Palinurin^118^ are worth mentioning (Figure 2). In particular tideglusib showed, in a first pilot study, a satisfactory safety profile and a significant improvement in cognition compared to placebo-patients.^119^ However, in a phase IIb trial, although it was well tolerated, no significant improvements were detected.^120^ Tideglusib has been also evaluated in trials for the treatment of other conditions, such as myotonic dystrophy,^121^ autism spectrum disorders,^122^ and progressive supranuclear palsy.^123^ In particular, in a phase II study in people with congenital and childhood-onset type 1 myotonic dystrophy, tideglusib at 1000 mg dose was well tolerated and improved multiple aspects of the symptomatology, such as neuromuscular, cognitive, and autism signs.^124^ In a phase II trial in patients with mild-to-moderate progressive supranuclear palsy, tideglusib, at 600 or 800 mg dose, was safe and generally well tolerated but it did not show any clinical efficacy.^123^ Another class of irreversible non-ATP competitive inhibitors is represented by halomethyl ketone derivatives that form a covalent bond with a key Cys199 located at the entrance of the ATP binding site.^125^

All the compounds classified as non-ATP competitive inhibitors usually establish weaker interactions with the enzyme, compared to ATP-competitive inhibitors, leading to a lower inhibition rate, which, in the case of GSK-3β, may be necessary to avoid toxicity. Due to GSK-3β involvement in several crucial biochemical pathways and its overactivation in pathological conditions, a weak inhibition able to bring enzymatic activity back to physiological levels may be sufficient to obtain the therapeutic effect without triggering toxic effects. Consequently, a weak to moderate inhibition of GSK-3β may be an optimal therapeutic approach. In this context, the main concerns are about the involvement of GSK-3β in glucose metabolism and in the Wnt signaling pathway. Indeed, many components of the Wnt/β-catenin pathway are involved in several cancers and GSK-3β inhibitors may be potentially oncogenic since GSK-3β suppresses tumor development.^126^ Indeed, this pathway affects several proto-oncoproteins, cell cycle regulators, and mediators of the epithelial to mesenchymal transition, which is critical for cancer metastasis. However, lithium has been shown to increase the level of β-catenin only in isolated cells while its long-term use in therapy has never been associated with an increased incidence of cancer.^110^

## 2 GSK-3β-Based Multitarget Ligands

In the past decades, anti-AD drug discovery has mainly focused on the Aβ cascade hypothesis although with disappointing results, as demonstrated by the recent failures of anti-Aβ antibody solanezumab and BACE-1 inhibitor verubecestat.^127^ However, other target-directed approaches have shown unsatisfactory results.^128^ There are many reasons for these failures. As previously discussed, a central point is represented by the fact that AD is a multifactorial disease driven by dysregulation of different but interconnected biochemical pathways. This recognition has led to a paradigmatic shift from the “one molecule–one target” to the “one molecule–multitarget” approach in drug discovery. Following this strategy, thousands of new chemical entities have been developed, called MTDLs, multitarget ligands (MTLs), hybrids or simply dirty drugs. As previously reported, without any doubt, anti-AD drug discovery is one of the main fields of application of the MTDLs design strategy.^5^ On the basis of the evidence pointing at GSK-3β as the functional link between Aβ and tau and due to its involvement in multiple pathways controlling crucial aspects of cell physiology, GSK-3β is gaining a lot of consideration as a drug discovery target. Indeed, promising MTDLs based on GSK-3β inhibitors are starting to appear. It is reported that a MTDL with higher possibility of success “*should be directed to networked targets whose connectivity has been proven*”,^2^ and GSK-3β fully satisfies this requirement representing a protein with plenty of tight connections with pathways and targets involved in AD pathogenesis. Furthermore, structural requirements necessary for GSK-3β inhibition are relatively simple and a good level of enzymatic inhibition may be achieved with low MW molecules. Herein, we will discuss some examples of GSK-3β-based MTDLs designed using the knowledge-based approach.

### 2.1 Dual GSK-3β/BACE-1 Inhibitors

In 2015, Cavalli, Bolognesi, and co-workers reported on one of the first examples of GSK-3β-based MTDLs as neuroprotective agents.^27^ In particular, as second target, the authors focused their attention on BACE-1. BACE-1 is a transmembrane aspartyl protease that is decisive for initiating Aβ generation that ultimately leads to the formation of Aβ plaques, one of the hallmarks of AD along with NFTs. Therefore, BACE-1’s critical role in regulating the first and rate-limiting step in Aβ production leads to the conviction that its inhibition may have a positive effect on Aβ plaques formation. Consequently, in recent years, many companies and academic laboratories have initiated programs to bring BACE-1 inhibitors into the clinic with disappointing results so far.^129^ Most of these BACE-1 inhibitors, such as lanabecestat, verubecestat, atabecestat, and elenbecestat, have reached late-phase clinical trials and are characterized by *in vitro* activity in the low nanomolar range.^130^

**Figure 3:**
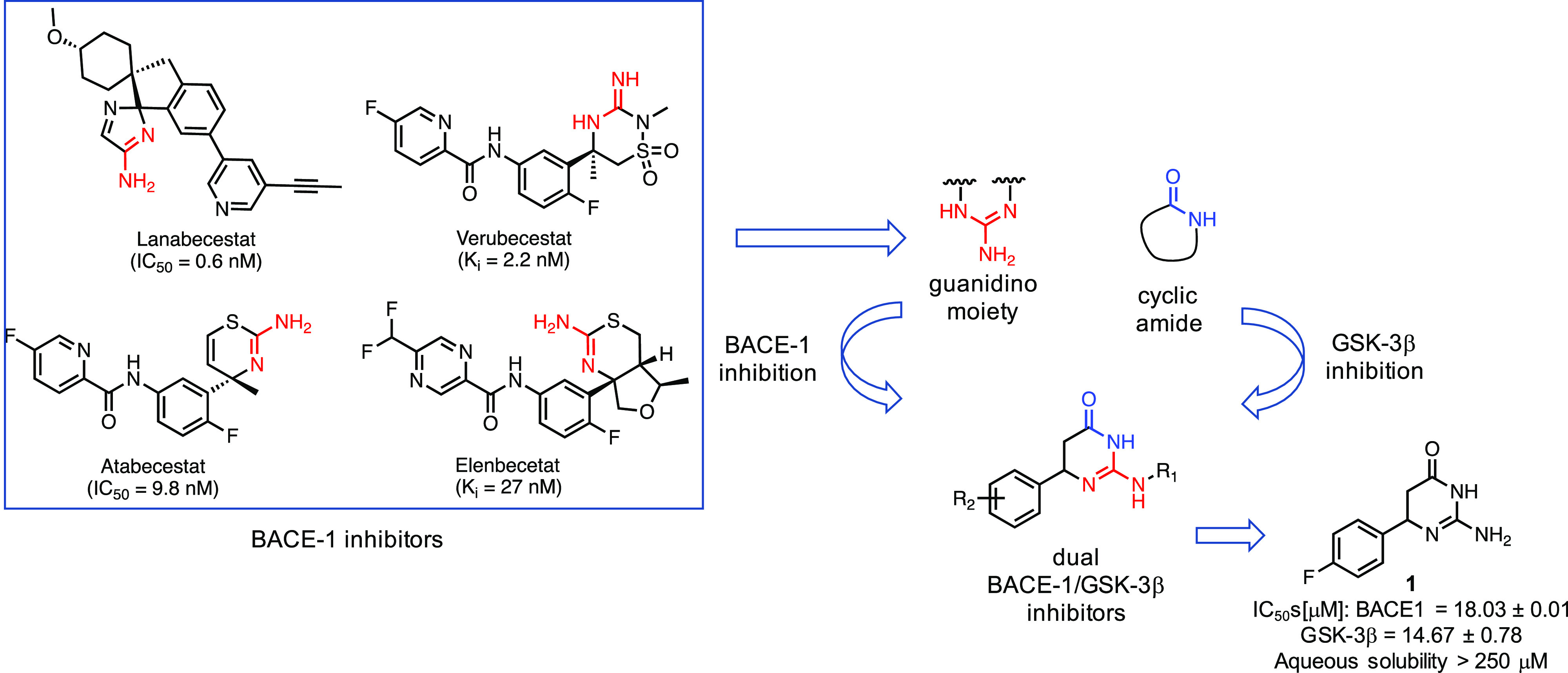
Design strategy leading to the discovery of dual GSK-3β/BACE-1 inhibitor **1**. Triazinones were obtained combining structural features responsible for GSK-3β and BACE-1 inhibition, a cyclic amide and a guanidino moiety, respectively. Compound **1** is the most interesting of the series with an IC_50_ in the micromolar range against both proteins.

On the basis of several pieces of evidence reporting that Aβ and tau are crucial partners that concurrently contribute to AD,^131^ the authors postulated that a molecular entity capable of concomitantly modulating targets that embody the crucial points of these two pathways may represent a true disease-modifying agent in AD therapy. Furthermore, several connections between GSK-3β and BACE-1 have been reported. For instance, (a) BACE-1 promotes Aβ formation, which in turn leads to an overactivation of GSK-3β, which consequently induces an increase in the formation of NFTs, inflammation, and cognitive impairment,^132^ and (b) GSK-3β regulates the activities of secretases.^92^ By exploiting a ligand-based approach, the authors identified and combined the pharmacophoric features responsible for GSK-3β and BACE-1 inhibition. In particular, they recognized (a) a guanidino moiety, present in several inhibitors, able to bind to the catalytic aspartic dyad of BACE-1 and (b) a cyclic amide, present in several ATP-competitive GSK-3β inhibitors, able to provide specific H-bonds networks. These two fragments have been combined to obtain a series of dual inhibitors 6-amino-4-substituted triazinone (Figure 3).^26,27^ Several analogs have been synthesized, most of which are characterized by a high water solubility. Compound **1** emerged as the most promising, as it showed moderate but balanced inhibitory profile (IC_50_: BACE-1 = 18.03 ± 0.01 μM, GSK-3β = 14.67 ± 0.78 μM). Moreover, it is characterized by a low MW and relative structural simplicity. Compound **1** reduced Aβ production in neuroglioma cell line expressing hAPP gene harboring Swedish mutation and did not show any significant toxicity in this cell line. In addition, compound **1** showed promising anti-inflammatory properties; in fact, it induced a reduction of nitrite formation at 10 μM and iNOS induction in astrocytes and microglia cells treated with LPS together with a switch in microglia from inflammatory M1 to anti-inflammatory M2 phenotype. Neurogenic properties of compound **1** have been observed in primary rat neural stem cells. Furthermore, pharmacokinetic analyses in mice showed that compound **1** had good oral bioavailability and BBB penetration. Indeed, after oral administration (10 mg/kg) *C*_max_ in plasma after 30 min was 665 ng/mL while 30 min after oral administration compound **1** reached the concentration of 0.613 ng/mL in 1 mL of brain homogenate.^27^

### 2.2 Dual GSK-3β/Tau Aggregation Inhibitors

**Figure 4:**
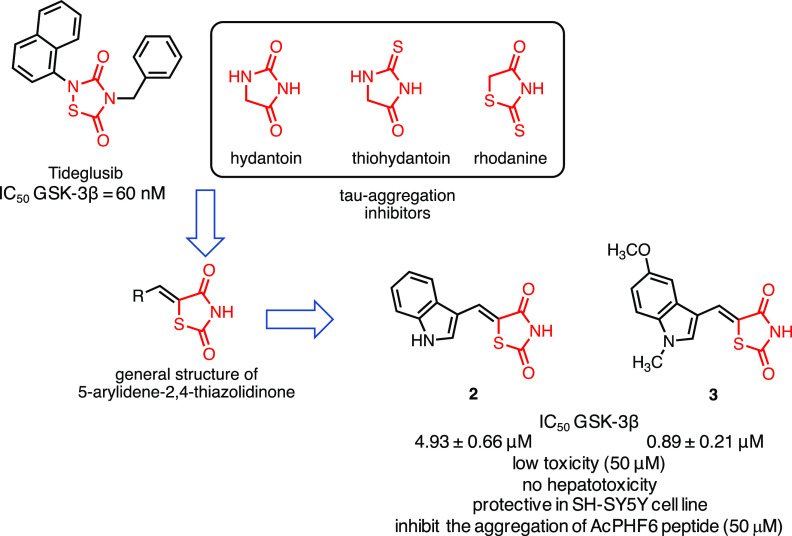
Design of dual GSK-3β/tau aggregation inhibitors. The 5-arylidene-2,4-thiazolidinedione has been designed taking into consideration that a five-member heterocycle is a moiety present in both GSK-3β and tau aggregation inhibitors and that a planar substituent in position 5 may increase both the selectivity toward GSK-3β and the interactions with the tau fibrils.

As previously discussed, GSK-3β is responsible for the hyperphosphorylation of tau protein increasing its propensity to aggregate leading to neuronal death. Bolognesi and co-workers designed a series of 5-arylidene-2,4-thiazolidinedione able to interfere in two crucial points of the tau network: (a) reduction of tau hyperphosphorylation and (b) inhibition of tau aggregation processes.^133^ The design strategy started with the observation that a five-member heterocycle is a common feature in different biologically active compounds, including both GSK-3β and tau aggregation inhibitors. For instance, tideglusib is a pentacyclic thiadiazolidinedione while thiohydantoin, hydantoin, and rhodanine are effective scaffolds to inhibit tau aggregation.^134^ However, the authors decided to explore the 2,4-thiazolidinedione fragment, which was never used before as GSK-3β or tau aggregation inhibitor, and to decorate it with an (hetero)aromatic moiety in position 5. Indeed, previous studies reported that the introduction of a 5-arylidene substituent in a series of 2-iminothiazolidin-4-one improved affinity and, more importantly, selectivity toward GSK-3β since such large substituents are not able to fit in similar regions of homologous kinases.^135^ Therefore, the substituent in position 5 has a double role: (a) it increases the volume and the interactions of the molecule with the aim of inducing selectivity toward other kinases since ATP binding pocket in GSK-3β is larger than its homologous counterparts,^135,136^ and (b) its planarity and aromaticity are crucial for the interaction with the tau fibrils (Figure 4). Among the different molecules synthesized, compounds **2** and **3** emerged as favorable lead compounds. In fact, they (a) selectively inhibited GSK-3β in an ATP-competitive manner, with an IC_50_ in the low micromolar range (4.93 ± 0.66 μM for **2** and 0.89 ± 0.21 μM for **3**), (b) showed PAMPA-BBB permeability, (c) were found to be not toxic in primary cultures of cerebellar granule neurons and human hepatoma cell line up to 50 μM, and (d) protect neuroblastoma SH-SY5Y cells from toxic insults induced by okaic acid. Compounds **2** and **3** have been evaluated for their ability to inhibit the aggregation of AcPHF6, a short peptide (_306_VQIVYK_311_) localized in the microtubule-binding moiety of tau protein that undergoes spontaneous fibrillation and has been proposed as a suitable model for screening of antiaggregants.^137^ Most importantly, compounds **2** and **3** inhibited the aggregation of AcPHF6 peptide (50 μM) at a concentration of 10 μM by stabilizing the peptide in a less fibrillogenic conformation.^133^

### 2.3 Dual GSK-3β/AChE Inhibitors

**Figure 5:**
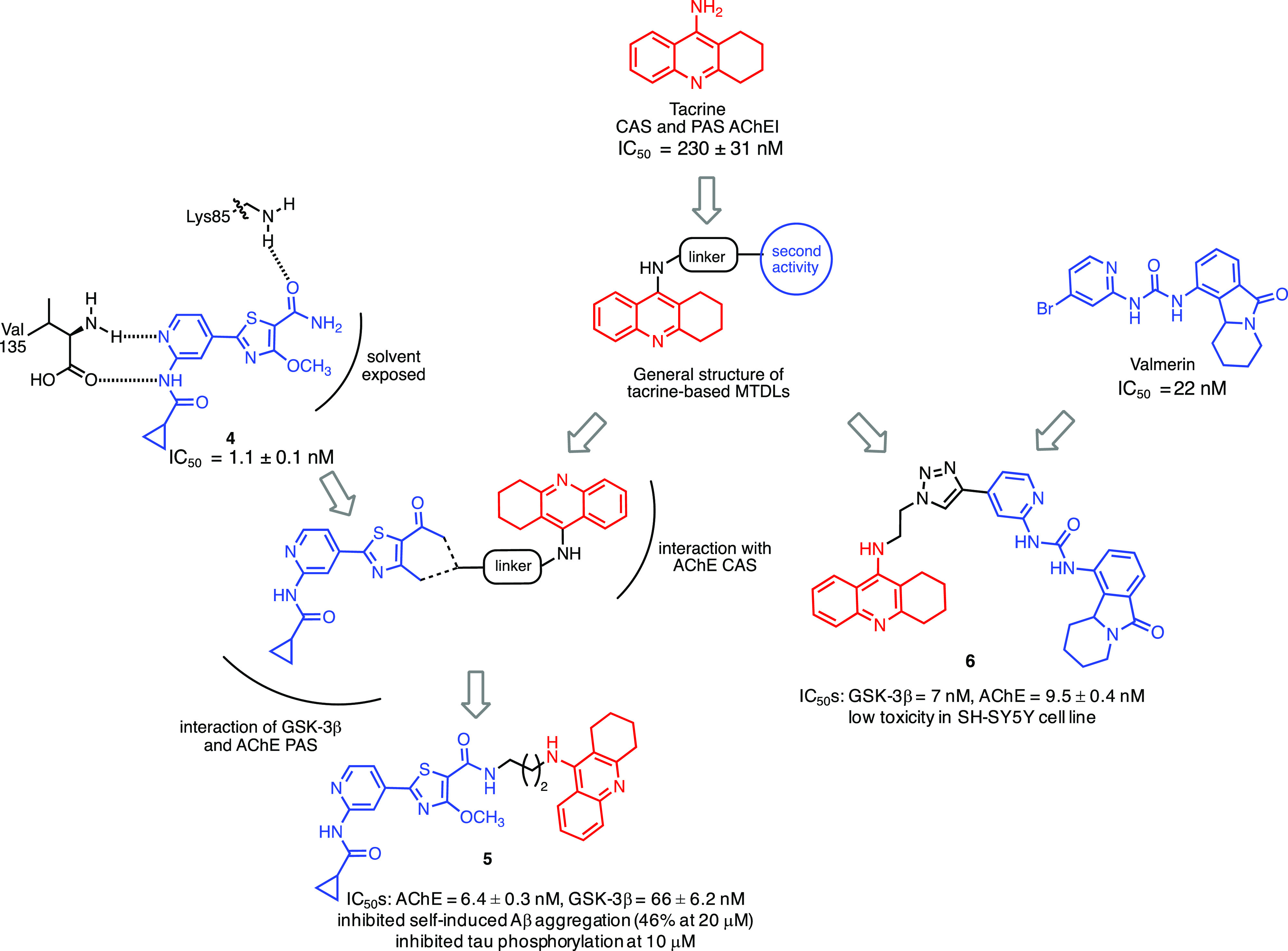
Design of dual GSK-3β/AChE inhibitors **5** and **6**. Tacrine has been extensively used to develop MTDLs by linking a structure responsible for inducing a second biological effect. In the cases reported in the figure, GSK-3β inhibitor **4** has been coupled to tacrine to obtain compound **5** by taking advantage of the amide in **4** localized in solvent-exposed portion of the molecule. In the second example, to obtain compound **6**, the structure of valmerin has been used as GSK-3β binding-fragment.

AChE is the enzyme responsible for the degradation of acetylcholine because of its catalytic site, and it is the target of one of the two classes of drugs currently available for AD treatment, although only as palliative. Several studies pointed out that AChE is also responsible for Aβ fibrils aggregation because of a secondary site located at the entrance of the enzymatic gorge, called peripheral anionic site (PAS).^138^ Therefore, blocking AChE may result in a double benefit: (a) improve cognition and (b) prevent the formation of Aβ plaques. During the rise of the MTDD design strategy, thousands of ligands, endowed with AChE inhibitory activity, have been coupled with a second pharmacophore to provide an additional biological activity such as inhibition of Aβ formation or aggregation, scavenging activity toward ROS and metal chelating properties. Most of these ligands were based on the structure of AChE inhibitor tacrine.^139^ In 2018, Sun and co-workers speculated that compounds able to simultaneously inhibit GSK-3β and AChE may represent suitable anti-AD agents due to their ability to interfere with NFTs and Aβ plaques formations.^140^ Hence, they reported the first class of dual GSK-3β/AChE inhibitors starting from the structure of tacrine, as AChE inhibitor, and a pyridothiazole as GSK-3β inhibitor **4** (Figure 5). The design of this new class of compounds was based on the observation that the carbonyl oxygen of compound **4**(141) establishes critical H-bonds with the Lys85 while the primary amide and the methoxy group are located in the solvent exposed site and, therefore, may be used as point to link the AChE binding fragment, namely, tacrine. Compound **5** showed good inhibitory activity against AChE and GSK-3β (IC_50_: AChE = 6.4 ± 0.3 nM, GSK-3β = 66 ± 6.2 nM) and self-induced Aβ aggregation (inhibitory rate 46% at 20 μM). Furthermore, it inhibited tau phosphorylation at Ser396 at 10 μM in mouse neuroblastoma N2a-Tau cells. In *in vivo* studies, compound **5** at 15 mg/kg ameliorated the cognitive disorders in scopolamine-treated ICR mice. Most importantly, compound **5**, contrary to tacrine, did not show any signs of hepatotoxicity, as confirmed by the reduction of alanine aminotransferase (ALT) and aspartate aminotransferase (AST).^140^

A second example of dual GSK-3β/AChE inhibitors appeared in 2019.^142^ In this investigation, tacrine and valmerin, a GSK-3β binding fragment, were linked through a triazole. Valmerin contains the tetrahydropyrido[1,2-*a*]isoindolone core and inhibits GSK-3β in the nanomolar range.^143^ Several analogs were synthesized, and compound **6** emerged as the most interesting since it showed a good inhibition profile (IC_50_: GSK-3β = 7 nM, AChE = 9.5 ± 0.4 nM) and low toxicity in different cell lines, including SH-SY5Y, and it was predicted to cross the BBB (Figure 5).^142^

### 2.4 Dual GSK-3β/Adenosine Kinase Inhibitors

**Figure 6:**
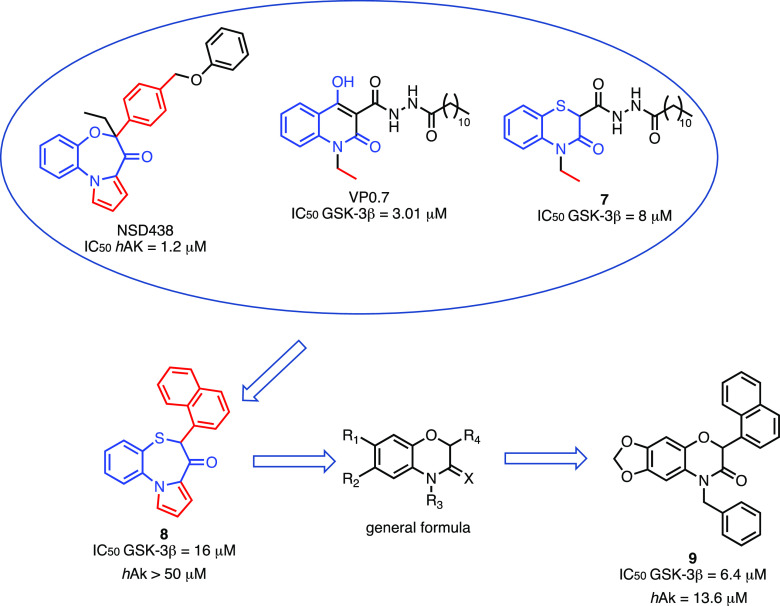
Design of dual GSK-3β/AK inhibitors. Compound **9**, able to bind to both GSK-3β and hAK in the micromolar range of concentrations, has been obtained through a series of modifications of compound **8**, developed by the same authors, that fails to bind to hAK.

Brogi and co-workers reported the first example of dual GSK-3β/adenosine kinase (AK) inhibitors as neuroprotective agents.^144^ Indeed, AK, as well as GSK-3β, is involved in oxidative stress modulation. In particular, AK phosphorylates nucleoside adenosine and displays protective effects reducing ROS levels by enhancing the activity of antioxidant enzymes, such as superoxide dismutase and glutathione peroxidase.^145^ Analyzing the structure of hAK inhibitors, such as NSD438^145,146^ and GSK-3β allosteric inhibitors, such as VP0.7^125^ along with compound **7**,^147^ the authors designed and synthesized compound **8** (Figure 6)^144^ able to inhibit GSK-3β but, unfortunately, with no activity on hAK at concentration lower than 50 μM. Starting from compound **8**, through ring contractions and groups replacement, the authors designed new benzoxazinones capable, in theory, of inhibiting both targets. Compound **9** was the most interesting of the series with an inhibitory profile in the micromolar range with no toxic effects in neuroblastoma cell line IMR 32 and capable of counteracting ROS formation.

### 2.5 GSK-3β Inhibitor/Metal Chelator

**Figure 7:**
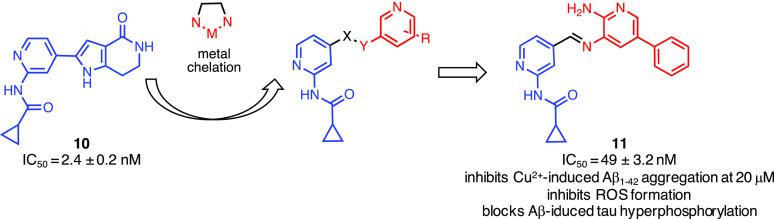
Design of dual GSK-3β inhibitors/metal chelators. The *N*-(pyridin-2-yl)cyclopropanecarboxamide, able to establish favorable interactions with GSK-3β, has been coupled with a substituted aminopyridine responsible for metal chelation.

Shi and co-workers focused their attention on metal dyshomeostasis.^148^ Changes in the transition metals, zinc, copper, and iron have been shown to affect the molecular mechanisms of the disease.^149^ High concentrations of metals have been found in Aβ plaques and were held responsible for accelerating the formation of Aβ oligomers.^150^ It was also reported that copper is able to contribute to ROS formation^151^ and oxidative stress was reported to affect tau protein phosphorylation and to significantly increase GSK-3β activity.^152^ To design this new class of multifunctional agents targeting GSK-3β and metal dyshomeostasis, the authors considered the structure of the GSK-3β inhibitor seen in compound **10**.^141^ The authors took into consideration the *N*-(pyridin-2-yl)cyclopropanecarboxamide, able to establish favorable interactions with the hinge region of GSK-3β (i.e., H-bonds with Val135), and replaced the pyrrolopyridinone with a substituted pyridine ring (Figure 7).^148^ An amine and an aromatic group were introduced as pyridine substituents aiming to establish additional interactions with GSK-3β and to chelate metals. Compound **11** was found to be a highly active GSK-3β inhibitor (IC_50_ = 49 ± 3.2 nM) chelating Al^3+^ and Cu^2+^, efficiently inhibiting Cu^2+^-induced Aβ_1–42_ (40 μM) aggregation at 20 μM, inducing disaggregation of Cu^2+^-induced Aβ_1–42_ aggregation and inhibiting ROS formations. Furthermore, compound **11** did not induce toxic effects in neuroblastoma SH-SY5Y cell lines, blocked Aβ-induced tau hyperphosphorylation at Ser396, and protected cells against toxicity induced by H_2_O_2_ (150 μM) at 10 μM.

### 2.6 Dual GSK-3β/Histone Deacetylase Inhibitors

**Figure 8:**
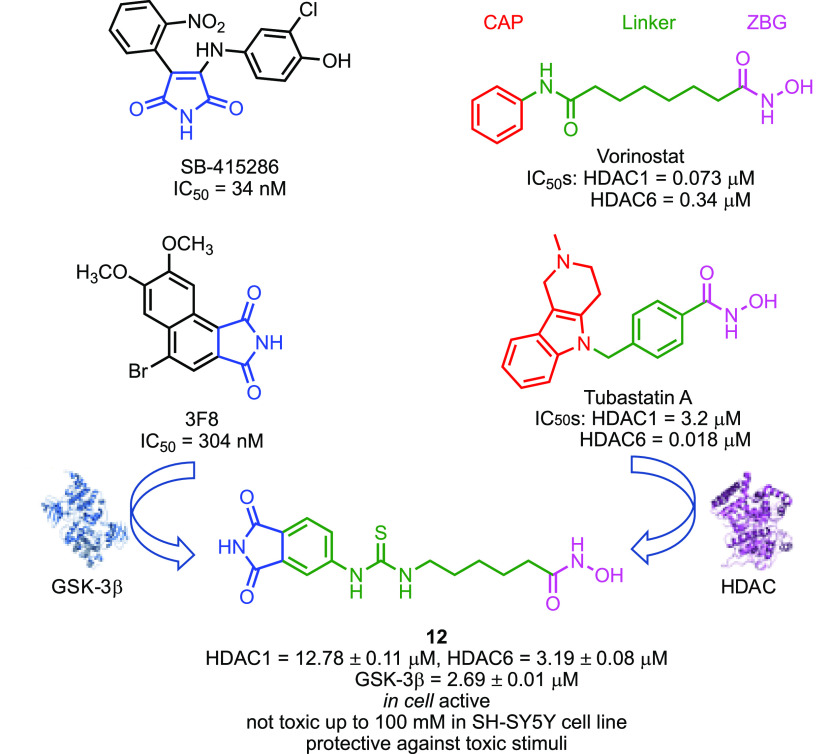
Design of dual GSK-3β/HDACs inhibitors. The phthalimide moiety, which interacts with the ATP-binding site of GSK-3β, and an hydroxamic acid, which chelates the Zn^2+^ located in the HDAC active site, were linked through an alkylthiourea chain.

In 2019, we focused our attention on histone deacetylases (HDACs), a prominent epigenetic target, and in particular on their role in neurodegeneration and its connections with GSK-3β.^153^ HDACs, together with the action of histone acetyltransferase, modulate both gene expressions and the functions of several non-histone proteins, such as tau, α-tubulin, and Hsp90. Due to the involvement of HDACs in neurodevelopment, memory formation, and cognitive processes, HDACs inhibitors (HDACIs) have been suggested as innovative agents for the treatment of neurodegenerative disorders such as AD.^154^ Although HDACIs have been used in clinical practice as anticancer agents, vorinostat, the prototype of HDACIs, has recently entered phase I clinical trial for AD. HDACIs have long been used to develop successfully MTDLs as antiproliferative agents. Only very recently, some examples of multiple drugs based on of HDACIs appeared in the literature.^155^ We planned to design a class of dual GSK-3β/HDACs inhibitors based on the strict connections existing between these two classes of enzymes; namely, (a) neurotoxic effects of HDAC1 depends on GSK-3β activity, and the block of such activity prevents HDAC1-induced cell death in cerebellar granule neurons, (b) GSK-3β and HDAC6 are found in the same protein complex where GSK-3β phosphorylates HDAC6, enhancing its activity (i.e., tau phosphorylation), and (c) combined inhibition of GSK-3β and HDACs induced synergistic neuroprotective effects compared to single-drugs combination treatment.^153^ To design this class of compounds, we combined in a single chemical entity the pharmacophoric groups responsible for binding to GSK-3β and HDACs. The HDACs pharmacophoric model is well-known and comprised a zinc binding group, able to chelate the Zn^2+^ ion located in HDACs active site, a linker, and a CAP group, usually an aromatic surface that interacts with the external surface of the enzyme. We choose a hydroxamic acid as zinc binding group and a phthalimide as CAP group, known to interact with the ATP-binding site of GSK-3β (Figure 8). From the *in vitro* evaluation against GSK-3β, HDAC1, and HDAC6, compound **12** emerged as the most interesting of the series. Compound **12** induced an increase in histone H3 and α-tubulin acetylation and blocked copper-induced tau hyperphosphorylation. In the latter case, the effect was more marked than that obtained with the combination of vorinostat and a pure GSK-3β inhibitor. Furthermore, it was shown to be nontoxic and protective against H_2_O_2_ and 6-OHDA stimuli in SH-SY5Y and in CGN cell lines, promoting neurogenesis and showing immunomodulatory effects.

## 3 Conclusions and Future Perspectives

The rise of polypharmacology and MTDLs strategy has revolutionized the design-paradigms well established in the medicinal chemistry community. Since the seminal papers by Morphy and Rankovic^3^ and Melchiorre and collaborators,^5^ we have witnessed an unprecedented explosion in the design of MTDLs directed toward neurodegenerative disorders, especially AD. However, on the basis of the most popular theories pursued by scientists to shed light on AD pharmacotherapy, the designed MTDLs were mainly centered on AChE and Aβ (both production and aggregation) inhibitors. Recently, some reports regarding MTDLs based on the tau hypothesis started to appear. This was mainly, but not only, because the Aβ theory, considered for years the cornerstone of AD, had disappointing results so far.^127^ Among the potential anti-tau targets, GSK-3β has been considered a primary focus due to its function as a link between tau and Aβ.^131^ Moreover, GSK-3β is a kinase critical in a multitude of CNS-specific signaling pathways and takes roles not only in tau- and Aβ-mediated toxicities but also in oxidative stress, inflammation, memory formation, and synaptic plasticity.^28^ Consequently, several studies have reported that GSK-3β inhibitors had efficiently antagonized neurodegeneration in different cell lines and *in vivo* models, some of which reach clinical trials.^52^ Furthermore, GSK-3β is networked with several other factors involved in AD, such as BACE-1, HDACs, etc.^27,153^ These important and confirmed connections make GSK-3β a key target for the design of more successful MTDLs even when characterized by lower affinity when compared to high-affinity single target-directed drugs.^2^

In this context, GSK-3β is a particularly appropriate target since (a) structure-based design strategies may be applied to design MTDLs given the availability of several X-ray solved protein–ligand structures, (b) the pharmacophoric model, at least for ATP-binding site inhibitors, is relatively simple and good inhibition levels may be achieved with high ligand efficiency leading, therefore, to more drug-like MTDLs, (c) strict kinase selectivity is not an absolute requirement since other kinases, such as CDK5 and protein-kinase A, are involved in tau phosphorylation and in critical CNS-signaling pathways,^65^ and (d) “soft” inhibition is required to obtain therapeutic effects and reduce the side ones. The latter point is very important since it is challenging to obtain high affinity target(s) while maintaining at the same time the structural requirements in terms of physical chemical characteristics. Higher GSK-3β activity is observed in neuropathological conditions, and approximately 20–25% inhibition is sufficient to produce therapeutic efficacy in CNS diseases.^156^ Therefore, IC_50_ in the low micromolar range of concentrations should be enough to obtain the desired pharmacological effects. Luckily, these levels of inhibition are not sufficient to impact other signaling pathways or target, such β-catenin, that may be responsible for side effects. This is confirmed by the long-used drug lithium whose employment is not associated with increased levels of tumorigenesis.^110^

On the basis of these considerations and on the above-reported examples of GSK-3β-directed MTDLs, we strongly support that GSK-3β could be used to design MTDLs with innovative mechanisms of actions. As briefly shown in this Miniperspective, the few examples of GSK-3β-based MTDLs are very promising in terms of anti-AD effects, toxicity, and physical chemical properties. As discussed, the design of MTDLs poses some challenges but we believe that the examples herein reported may induce a shift from an AChE-centric to a tau-centric paradigm leading to a new “gold rush” in the design of anti-AD MTDLs.

## Abbreviations Used

AChE: acetylcholinesterase
AD: Alzheimer’s disease
AK: adenosine kinase
APP: amyloid precursor protein
Aβ: β-amyloid
ATP: adenosine triphosphate
BACE-1: β-secretase
CDK5: cyclin dependent kinase 5
CREB: cAMP response element-binding protein
DAPK1: death-associated protein kinase 1
GS: glycogen synthase
GSK-3β: glycogen synthase kinase 3β
HDAC: histone deacetylase
HSF1: heat shock factor 1
Hsp90: heat shock protein 90
HTS: high throughput screening
LTP: long-term potentiation
LTD: long-term depression
MAPK: p38α mitogen-activated protein kinase
MTD: multitarget drug
MCI: mild cognitive impairment
MTDL: multitarget-directed ligand
MW: molecular weight
NMPA: China’s National Medical Product Administration
NFT: neurofibrillary tangle
PAS: peripheral anionic site
PKA: cyclic AMP-dependent protein kinase
PKB/Akt: protein kinase B
PKC: atypical protein kinase C
PPI: protein–protein interaction
PS1: presenilin 1
ROCK1: Rho-associated protein kinase 1
